# How Flaviviruses Activate and Suppress the Interferon Response

**DOI:** 10.3390/v2020676

**Published:** 2010-02-23

**Authors:** Jorge L. Muñoz-Jordán, Brenda L. Fredericksen

**Affiliations:** 1 Molecular Diagnostics and Research Laboratory, Centers for Disease Control and Prevention, Division of Vector Borne Infectious Diseases, Dengue Branch, 1324 Calle Cañada, San Juan, PR 00920, Puerto Rico; 2 Department of Cell Biology and Molecular Genetics and Maryland Pathogen Research Institute, University of Maryland, MD 20742, USA; E-Mail: bfreder@umd.edu

**Keywords:** flavivirus, dengue, West Nile, interferon

## Abstract

The flavivirus genus includes viruses with a remarkable ability to produce disease on a large scale. The expansion and increased endemicity of dengue and West Nile viruses in the Americas exemplifies their medical and epidemiological importance. The rapid detection of viral infection and induction of the innate antiviral response are crucial to determining the outcome of infection. The intracellular pathogen receptors RIG-I and MDA5 play a central role in detecting flavivirus infections and initiating a robust antiviral response. Yet, these viruses are still capable of producing acute illness in humans. It is now clear that flaviviruses utilize a variety of mechanisms to modulate the interferon response. The non-structural proteins of the various flaviviruses reduce expression of interferon dependent genes by blocking phosphorylation, enhancing degradation or down-regulating expression of major components of the JAK/STAT pathway. Recent studies indicate that interferon modulation is an important factor in the development of severe flaviviral illness. This suggests that an increased understanding of viral-host interactions will facilitate the development of novel therapeutics to treat these viral infections and improved biological models to study flavivirus pathogenesis.

## 1. Detection of Flaviviruses by the Host Cell

**Figure 1.**
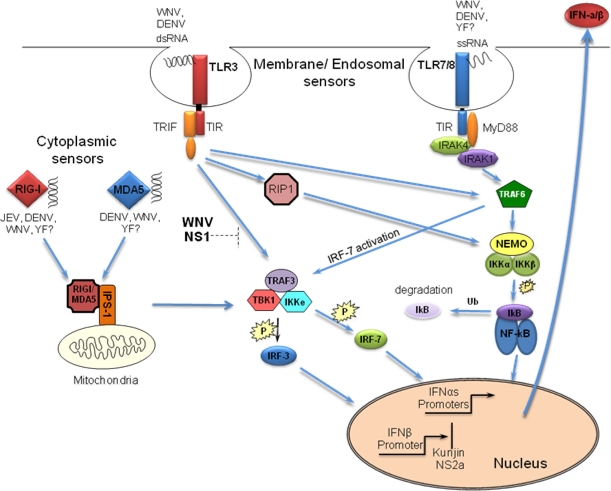
PRRs involved in detecting flaviviruses. Dashed line indicates cell type and/or context-dependent blockade of pathway.

Mammalian cells utilize specialized cellular proteins termed pathogen-recognition receptors (PRRs) to sense invading pathogens. These proteins function by recognizing specific pathogen-associated molecular patterns (PAMPs) produced during the course of infection. Two classes of PRRs, the toll-like receptors (TLRs) and the retinoid-inducible gene I (RIG-I)-like receptors (RLRs), are essential for responding to viral infection [1]. The various PRRs recognize different viral structural and/or functional features; nonetheless, they all function to initiate signaling cascades that result in the activation of transcription factors critical for the onset of the type 1 interferon (IFN-α/β) response. Overexpression studies *in vitro* as well as targeted gene depletion *in vivo* suggest that both the TLR and RLR pathways play vital roles in detecting and responding to flavivirus infections (Figure 1). However, the specific PRRs involved in mediating the antiviral response are likely to be virus- and cell-type specific.

## 2. Activation of RLR by Flaviviruses

The RLR family members RIG-I and MDA5 are ubiquitous cytosolic proteins that mediate the host’s intracellular antiviral response to viral infection. These cytoplasmic receptors are essential for detecting RNA viruses in most cell types [2–5]. RIG-I and MDA5 both contain two N-terminal caspase recruitment domains (CARD) followed by a single DExD/H box RNA helicase domain. Binding of viral PAMPs to the helicase domain is postulated to induce conformational changes that allow these RLRs to interact with the downstream adaptor protein IPS-1/MAVS/CARDIF *via* their CARD domains. These interactions initiate a signaling cascade, resulting in the activation of transcription factors such as IRF-3, IRF-7 and NFκB, which are required for the induction of IFN-α/β and the establishment of an antiviral state within the cell. Several groups have demonstrated that RIG-I preferentially recognizes single-stranded RNA (ssRNA) molecules containing free terminal 5′ triphosphates [6–9]. However, a recent study by Kato *et al.* demonstrated that RIG-I and MDA5 interact with double-stranded RNAs (dsRNA) in a length-dependent manner, regardless of 5′ end modifications [10]. Short dsRNA molecules were shown to bind to and activate RIG-I while long dsRNAs functioned solely as agonists of MDA5. These studies indicate that RIG-I recognizes the 5′ triphosphates present on uncapped termini of viral genomes and dsRNA produced during the course of infection, while MDA5 recognizes long dsRNA viral genomes or long duplex RNAs produced during genome replication.

RIG-I has been shown to be involved in sensing every member of the flavivirus genus examined to date. Stimulation of the IFN-α/β promoter in response to Japanese encephalitis virus (JEV) infection was reduced in cells overexpressing a dominant negative form of RIG-I and was completely lacking in mouse embryo fibroblasts (MEFs) recovered from RIG-I^−/−^ mice [5,11]. Furthermore, RIG-I-deficient mice exhibit a marked decrease in serum IFN-α/β levels and an increased susceptibility to JEV compared to wild type control mice, while deletion of MDA5 has no affect [5]. This suggests that RIG-I, but not MDA5 signaling pathways are involved in initiating the antiviral response to JEV. In contrast, disruption of RIG-I signaling does not ablate the induction of antiviral programs in response to dengue Virus (DENV) and West Nile virus (WNV) infection [12–14]. In the case of WNV, the onset of the innate antiviral response was merely delayed in RIG-I^−/−^ cells compared to wild type controls. This suggests that the RIG-I pathway mediates the initial activation of the antiviral response to WNV, though distinct secondary pathways are also clearly involved. Nonetheless, WNV replication is enhanced in the absence of RIG-I, indicating that this pathway plays a critical role in constraining WNV. The fact that cells respond to WNV and DENV in the absence of RIG-I suggests that other PRRs are also involved in the detection of these viruses. Several lines of evidence indicate that MDA5 functions as the secondary receptor for sensing both WNV and DENV. As with RIG-I-deficient cells, MDA5^−/−^ MEFs were shown to retain the ability to respond to WNV and DENV infection [12,14]. In addition, disruption of both the MDA5 and RIG-I signaling pathways abrogated the response to WNV and DENV, indicating that both viruses trigger RIG-I and MDA5-dependent responses [12,14]. This is further supported by the observation that IPS-1 null MEFs were refractory to WNV and DENV-mediated activation of IRF-3. Additionally, RIG-I and MDA5 expression is upregulated in WNV and DENV-infected MEFs as well as DENV-infected muscle satellite cells, monocytes, B cells and dendritic cells (DCs) [12,14]. Collectively, the evidence indicates that both RIG-I and MDA5 play important roles in initiating and sustaining the antiviral response to WNV and DENV.

The role of the RLR system in controlling yellow fever virus (YFV) has yet to be examined. However, both RIG-I and MDA5 expression was upregulated in peripheral blood mononuclear cells (PBMCs) recovered from individuals vaccinated with YF-17D [15], suggesting a possible role for both of these PRRs in responding to YFV *in vivo*. Combined, these studies indicate that RIG-I is involved in sensing all flavivirus infections; however, MDA5’s role is virus-dependent.

## 3. Activation of TLRs

Members of the TLR family are evolutionarily conserved transmembrane molecules that are expressed on the cell surface or within endocytic vesicles in a cell type-dependent manner [2,16]. Expression of the various TLRs is typically restricted to specific subtypes of immune cells, suggesting that these receptors play distinct roles in triggering a response to an invading pathogen [17]. Detection of PAMPS is mediated by the leucine rich repeats located in the ectodomain of the TLR. Thus, TLRs are restricted to the detection of either extracellular or vesicle-bound PAMPs. Binding of extracellular ligands to the TLRs initiates a signal transduction cascade through a Toll/IL-1 receptor (TIR) homologous domain located in the cytoplasmic region of the protein. The adaptor protein MyD88 mediates the signaling pathways of all TLRs except TLR3, which utilizes TIR-domain-containing adaptor-inducing interferon-β (TRIF) instead. Cellular localization of TLR3 and TLR7 is cell type-dependent. Human fibroblasts express TLR3 and TLR7 on the cell surface. However, these TLRs are localized to intracellular compartments of the endocytic pathway in cells of immune origin [2,16]. TLR3 and TLR7 function as a broad sensor of dsRNA and ssRNA, respectively. However, TLR7 response to ssRNAs is enhanced by higher order structures within viral RNA [18–20]. As with the RLRs, stimulation of the TLR pathways results in a multivalent signaling cascade that leads to the production of IFN-α/β and inflammatory cytokines, which in turn stimulates maturation of DCs and the establishment of an antiviral response [21].

The involvement of the various members of the TLR system appears to be virus dependent. Both TLR3 and TLR7 have been shown to be involved in sensing DENV and WNV. Silencing of TLR3 expression in human monocyte cell lines altered cytokine production in response to DENV infection [22]. Additionally, overexpression of TLR3 enhanced cytokine production and inhibited DENV replication. This suggests that TLR3 may be an important component of the antiviral response to DENV. However, the observation that DENV infection failed to induce cytokine production in bone marrow-derived macrophages from MyD88 null mice suggests that additional TLRs are also involved in sensing this virus [23]. This is further supported by the fact that TLR7 specific inhibitors attenuated IFN-α/β production by plasmacytoid dendritic cells (pDCs) in response to DENV [20]. Furthermore, treatment of monocytes and DCs with bafilomycin A, a vacuolar H+-ATPase inhibitor, suppressed DENV-induced production of IFN-α/β and IL-8, indicating that endosomal acidification is necessary for the innate detection of this virus [20,22]. Since TLR7 signaling and viral entry into the cells require acidification of endocytic vesicles, it has been proposed that detection of DENV by TLR7 is coupled to viral fusion and uncoating [20].

TLR3 and TLR7 have also been implicated in WNV infections [24–26], though the role of these TLRs in WNV-mediated pathogenesis remains controversial. Wang *et al.* observed that WNV virulence was attenuated in TLR3^−/−^ mice [24], despite increased viremia. The authors proposed that the enhanced virulence in wild type mice was due to an increase in the permeability of the blood-brain barrier caused by induction of an inflammatory response by TLR3. Therefore, stimulation of the TLR3 pathway by WNV *in vivo* leads to increased pathogenesis rather than protection. In contrast, a recent study re-examining the pathogenesis of WNV in TLR3-deficient mice reported an increase in the susceptibility to WNV in these mice [25].

It has also been demonstrated that mice deficient in either MyD88 or TLR7 exhibit increased viremia and enhanced susceptibility to WNV infection when challenged through an intraperitoneal route [24]. Additionally, WNV-infected TLR7-deficient mice exhibited increased systemic levels of the proinflammatory cytokines IFN-α, IFN-β, IL-6, IL-1b and TNF-α compared to wild type control mice. However, decreased levels of IL-12 p35 and IL-23 p19 were detected in the brain of TLR7^−/−^ mice infected with WNV. The reduction in IL-23 expression corresponds with a decrease in infiltration of peripheral immune cells into infected target organs in TLR7^−/−^ mice challenged with a lethal dose of WNV. This suggests that the reduced survival of WNV-infected TLR7^−/−^ mice is due to a diminished ability to trigger migration of the immune cells responsible for neutralizing and clearing the infection to the proper locations.

More recently it has been suggested that TLR7 may in fact play a role in promoting WNV infection [26]. Reduced numbers of Langerhans dendritic cells (LC) were observed in the epidermis of wild type but not TLR7^−/−^ mice following cutaneous challenge with WNV. This suggests that the TLR7 response may stimulate LC migration to the draining lymph nodes, thereby counteracting the protective function of the TLR7 response by promoting dissemination of WNV to peripheral tissues. However, the survival rates of wild type and TLR7^−/−^ mice infected with WNV either intradermally or by infected mosquito feeding were not significantly different. These data suggest that both TLR7 and TLR3 contribute to the antiviral response to WNV, though the exact role of these pathways in WNV-mediated pathogenesis remains to be determined.

TLRs have also been implicated in the activation of DCs by YF-17D [27]. DCs recovered from MyD88, TLR2, TLR7 and TLR9 -deficient mice all exhibited reduced levels of cytokine production in response to YF-17D. In addition, human fibroblasts stably transfected with TLR8 and an NF-κB luciferase reporter responded more robustly to YF-17D, suggesting that this TLR8 is also capable of detecting the virus [27].

Combined, these recent studies indicate that the TLR system plays a role in stimulating the antiviral response to YFV, DENV and WNV but not to JEV. TNF-α/β levels in JEV infected DCs were unaffected by ablation of MyD88 and more importantly depletion of MyD88 had no effect on susceptibility to JEV *in vivo* [5,23]. In sum, multiple PRR are clearly involved in the initiation of the antiviral response to most flaviviruses; however the pathways engaged during infection are virus dependent.

## 4. Evasion of the Host Recognition

The TLR and RLR signaling cascades converge at the point of activation of the latent transcription factors IRF-3 and NFκB. Activation of these transcription factors is critical for the rapid establishment of an antiviral state within the cell and induction of IFN-α/β. Many viruses induce activation of IRF-3 within 3–6 h post-infection [28–32]. However, pathogenic strains of WNV fail to stimulate the IRF-3 transcriptional activity until approximately 12–16 h post-infection, with maximal activation occurring much later [33]. This allows WNV to replicate to high levels prior to the induction and release of IFN-α/β, which provides two advantages to the virus. First, WNV is able to rapidly spread to neighboring uninfected cells, thereby outpacing the paracrine antiviral effects of IFN-α/β. Second, accumulation of viral proteins capable of attenuating the Janus kinase and signal transducers and activators of transcription (JAK/STAT) signal transduction pathway may render the infected cell refractory to the antiviral activity of IFN-α/β. The mechanism by which WNV avoids detection by PRRs early in infection remains to be determined. One possible explanation is that high levels of the WNV agonist(s) are required for efficient activation of IRF-3, such that activation does not occur until sufficient levels of the viral agonist(s) have accumulated. Alternatively, WNV may have evolved to specifically mask viral agonist(s) produced early in infection; thus blocking their accessibility to PRRs until the virus has established a productive infection. Additionally, expression of the WNV NS1 protein individually or in the context of a replicon has been shown to impede TLR3-mediated activation of IRF-3 and NFκB in HeLa and 293 cells overexpressing TLR3. However, many cell lines infected with WNV remain responsive to soluble, intracellular and virally encoded forms of dsRNA ligands [13]. This suggests that the WNV NS1-imposed blockage of TLR3 may be cell type and/or context-dependent (Figure 1).

Downstream of the PRRs, the transcription factor IRF-3 plays a pivotal role in controlling WNV replication and spread both *in vitro* and *in vivo* [34,35]. Mice lacking IRF-3 exhibited increased viral levels in the blood, peripheral organs and central nervous system (CNS). Furthermore, the absence of IRF-3 also resulted in an expanded tissue tropism, earlier entry into the CNS and ultimately increased susceptible to WNV infection. Yet, systemic levels of IFN-α/β in mice were unaffected by the ablation of IRF-3. This suggests that the protective effect of IRF-3 was due to antiviral actions of direct target genes. Indeed, macrophage and cortical neuronal cells derived from IRF-3 deficient mice confirmed that IRF-3 signaling triggers IFN-dependent and independent pathways important for controlling WNV replication. This suggests that WNV is sensitive to the antiviral actions of the IRF-3 pathway and that the ability to delay the activation of this arm of the host response may be central to WNV’s ability to achieve high levels of replication *in vitro* and *in vivo* [34,35].

## 5. Suppression of the IFN-α/β Signaling by Flaviviruses

Activation of the transcription factors IRF-3, IRF-7 and NF-κB through either the TLR or RLR systems results in the production of IFN-α/β, which is essential for the amplification of the response to the invading flaviviruses. Binding of secreted IFN-α/β to the IFN-α/β receptor on the surface of infected cells triggers the activation of the JAK/STAT signal transduction pathway. This in turn results in the stimulation of hundreds of promoters containing IFN-α/β-stimulated regulatory elements (ISRE), thus driving the expression of the wide variety of interferon stimulated genes (ISGs) that are responsible for establishing the antiviral state within the cell [36,37].

Accumulating evidence suggests that IFN-α/β has the potential to play an important role in inhibiting flavivirus replication. Pretreatment of human hepatoma cells with IFN-α/β results in inhibition of DENV replication. This inhibition is retained even when DENV RNA is transfected directly into cells, indicating that IFN-α/β affects post-entry steps of viral replication [38]. Likewise, WNV has been shown to be sensitive to antiviral effects of IFN-α/β *in vitro* [39–43]. Pretreatment of human and mouse cells with IFN-α/β inhibited WNV replication, though the magnitude of the sensitivity of WNV to IFN-α/β was cell line and strain-dependent [39,40,42].

The importance of the IFN-α/β pathway in controlling flavivirus infection has also been demonstrated *in vivo*. Ablation of the IFN-α/β receptor or the JAK/STAT signaling pathway increased susceptibility to DENV, WNV and JEV infection [40,43–47]. While IFN pretreatment protected animals against lethal challenges with WNV and St. Louis encephalitis virus [48].

DENV and WNV infections have been shown to induce the IFN-α/β response both *in vitro* and *in vivo*. Global and targeted gene expression profiling using various cell lines confirms the upregulation of IFN-α/β as well as downstream ISGs in response to either DENV or WNV infection [14,35,49–51]. Induction of IFN-α/β has also been detected in mice infected with WNV [25,34,43,52]. Additionally, high levels of IFN-α/β are present for long periods of time in pediatric dengue patients after defervescence; and differential global gene expression profiling has shown that key mediators of the IFN-dependent antiviral response are upregulated in patients [49,53–55].

However, WNV and DENV are still capable of establishing productive infections despite the host’s ability to stimulate a robust IFN-α/β response to these viruses. Recent evidence suggests that severe disease associated with DENV and WNV infections correlates with their ability to counteract the IFN-α/β response [40]. The highest amounts of IFN-α/β detected in acutely ill dengue patients occur very early in infection, with IFN-α/β levels decreasing with disease progression [56]. Furthermore, DENV infection has been demonstrated to stimulate maturation of infected DCs and uninfected bystander cells, which leads to a robust induction of IFN-α/β, TNFα and significant pro-inflammatory cytokines [57,58]. However, the activation of infected DCs was blunted compared to uninfected cells. These findings suggest that DENV blocks or circumvents the IFN-α/β response. Thus, allowing the virus to propagate in the presence of IFN-α/β levels that would otherwise be a sufficient to impair its replication. The first experimental confirmation of DENV’s ability to block the IFN-α/β response was provided by Diamond and Harris [38], who demonstrated that a short incubation of cells with IFN-α/β prior to infection was required to completely inhibit viral replication. Additionally, DENV-encoded proteins are directly implicated in IFN-α/β antagonistic functions. The DENV-encoded proteins NS2A, NS4A and NS4B, expressed separately in human alveolar basal epithelial cells (A549), enhanced replication of IFN-α/β-sensitive viruses in the presence of IFN-α/β, and NS4B strongly inhibited IFN-α/β stimulation of ISRE promoter [59]. Co-expression of NS2A, NS4A and NS4B completely ablated IFN-α/β signaling, suggesting that these three proteins have a synergistic inhibitory effect on the JAK/STAT signaling pathway [59,60].

**Figure 2.**
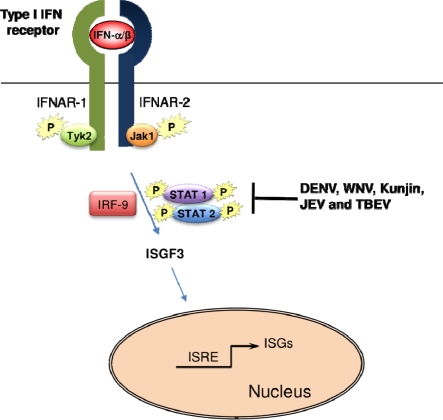
Suppression of the IFN-α/β signaling by flaviviruses.

Co-transfection of NS4A/B together with NS2B/3 resulted in the cleavage of NS4A and NS4B and levels of IFN-α/β inhibition comparable to those obtained by co-transfection of the individual NS4A and NS4B proteins, indicating that the proteolytic processing of the NS4A/B region is needed for anti-interferon function [59]. Correct targeting of NS4B to the ER is also required for its anti-interferon activity, as deletion of the 2K segment without replacement by another signal peptide resulted in impairment of IFN-antagonistic function [60]. Additionally, transfection experiments show that cytoplasmic segments between the first and second transmembrane regions of NS4B are required for IFN-α/β antagonism. These experiments indicate that non-structural protein segments of DENV interact with components of the IFN-α/β pathway [59,61,62]; however, such interactions remain to be more precisely defined. The ability of NS4B to impair JAK/STAT signaling in Vero cells is conserved in both YFV and WNV, possibly indicating a consensus mechanism to block this pathway in mosquito-borne flaviviruses [40,41,60,63]. Indeed, both DENV and WNV have been shown to block JAK/STAT signaling by disrupting phosphorylation of STAT-1 (Figure 2) [40,41,60,63].

Recent evidence suggests that DENV encodes additional mechanisms to block IFN-α/β. STAT2 levels were shown to be reduced in K562 (human chronic myeloid leukemia) and THP-1 (human monocytic) cell lines stably transfected with DENV replicons expressing all DENV non-structural proteins [64]. Furthermore, the reduction in STAT2 expression was shown to be due to NS5-mediated degradation [65,66]. As with NS4B, appropriate folding and posttranslational cleavage steps of NS5 are required for antagonism of the IFN-α/β pathway. While DENV NS5 alone is capable of binding STAT2, its ability to target STAT2 for degradation requires the presence of a protease cleavage signal upstream of the N terminus of NS5; thus mirroring the NS5 processing that occurs in the context of the DENV polyprotein during a natural infection [65]. Reduced levels of STAT2 and inhibition of STAT1 phosphorylation have also been correlated with the down-regulation of Tyk2 [67,68]. This places the interactions between DENV non-structural proteins and the IFN-α/β system in upstream components of the JAK/STAT signaling pathway. Given that DENV NS5 binds STAT2, it is tempting to speculate that NS4B may be involved in Tyk2 down-regulation; however, this remains to be experimentally confirmed.

Unlike DENV, expression of JEV or Tick-borne encephalitis virus (TBEV) NS5 alone is sufficient to inhibit IFN and mimic the effect observed with JEV or TBEV infection [69]. The inhibition of IFN signaling by JEV and TBEV NS5 homologues does not involve binding to the STATs but rather upstream events in the IFN pathways. In the case of tick-borne flaviviruses, the minimal requirement for this function has been ascribed to residues in two noncontiguous sequences of the RNA-dependent RNA polymerase region of NS5, which appears to come together in the tridimensional structure of this protein. Whether the expression of these proteins in the context of a cleaved precursor would confer additional functions has not been examined. Indeed, the NS5 protein of Kunjin virus, an Australian substrain of WNV, requires expression within the context of the NS1-5 region for efficient *trans-*complementation of a self replicating Kunjin minigenome [70]. Expression of the Kunjin NS5 protein alone results in a 100-fold decrease in replication activity, suggesting that appropriate cleavage of NS5 is required in order to achieve optimal catalytic activity. This raises the possibility that other flaviviral proteins may also require proper cleavage in order to display their full anti-interferon functions.

Viruses often encode complex, redundant mechanisms to antagonize the antiviral response of the host. Many viruses circumvent the IFN-α/β response by preventing the expression of IFN. Hepatitis C virus blocks IFN-α/β production by cleaving the cytoplasmic domain of the RLR signaling adaptor molecule IPS-1. As a consequence, IPS-1 loses its essential association to the mitochondria, which precludes effective binding with RIG-I and MDA5 and thereby abolishes RLR-mediated induction of IFN expression [71,72]. Although inhibition of IFN-α/β expression by DENV proteins has not been observed, this possibility cannot be ruled out. The blocking of IFN-α/β signaling, and not IFN-α/β expression, is supported by the fact that DENV NS4B protein specifically blocks signaling through the JAK/STAT pathway [60]. However, the IFN response undergoes auto-amplification as an infection progresses, and inhibition of the IFN-α/β signaling will in itself result in reduced IFN-α/β production. For example, TLRs and RLRs expression is upregulated by IFN-α/β. Therefore inhibition of the JAK/STAT pathway renders the cell less responsive to viral infection, and in so doing it reduces the expression of IFN-α/β. Further investigation will more precisely elucidate the extent to which the IFN-α/β network is antagonized by DENV. On the other hand, Kunjin virus has been shown to regulate the expression of IFN-α/β. A single amino acid substitution in the NS2A protein of a Kunjin virus resulted in increased levels of IFN-α/β expression both *in vitro* and *in vivo* and a corresponding decrease in virulence in mice [73]. However, the mechanism by which the NS2A protein of Kunjin virus controls the level of IFN-α/β expression remains to be determined.

## 6. Conclusions

The TLR and RLR systems combat invading pathogens by (1) reprogramming the cell’s gene expression profile to establish an antiviral state and (2) inducing the expression of pro-inflammatory and antiviral cytokines in order to limit the viral spread. However, viruses have evolved multiple processes to escape the innate antiviral response. In case of flaviviruses, we are just beginning to recognize how intricate and redundant these mechanisms are. The flaviviral non-structural proteins clearly play an important role in attenuating signaling through the JAK/STAT pathway. However, there is much still to learn about the race between flavivirus replication and the antiviral response at the molecular level. Further studies will be required to tease apart the viral-host interacts that ultimately determine the disease outcome.

## Abbreviations

CARDIF: Caspase recruitment domain adaptor inducing IFN-β
CARD: Caspase recruitment domains
CNS: Central nervous system
DCs: Dendritic cells
DENV: Dengue virus
dsRNA: Double-stranded RNA
ER: Endoplasmic reticulum
IKK: Inhibitor of kappa kinase
Iκβ: Inhibitor of NF- κβ
IL: Interleukin
IFN-: Interferon
IFN-AR: Interferon-α/β receptor
IPS-1: Interferon-beta promoter stimulator
IRAK: Interleukin (IL)-1 receptor–associated kinase
IRF: Interferon regulatory factor
ISGF: Interferon-stimulated gene factor
ISGs: Interferon stimulated genes
ISRE: Interferon-stimulated response element
JAK: Janus kinases
JEV: Japanese encephalitis virus
LC: Langerhans dendritic cells
MAVS: Mitochondrial anti-viral signaling protein
MDA5: Melanoma differentiation-associated gene 5
MEFs: Mouse embryonic fibroblasts
MyD88: Myeloid differentiation primary response gene (88)
NEMO: Nuclear factor kappa B essential modifier
NF-κβ: Nuclear Factor-Kappa Beta
NS: Nonstructural
PAMPs: Pathogen-associated molecular patterns
PBMC'S: Peripheral blood mononuclear cells
pDCs: Plasmacytoid dendritic cells
PRRs: Pathogen-recognition receptors
RIG-I: Retinoic acid inducible gene I
RIP1: Receptor interacting protein 1
RLRs: RIG-1-like receptors
ssRNA: Single-stranded RNA
STAT: Signal transducers and activators of transcription
TBEV: Tick-borne encephalitis virus
TBK1: TANK-binding kinase 1
TIR: Toll/IL-1 receptor
TLRs: Toll-like receptors
TNF: Tumor necrosis factor
TRAF: TNF receptor associated factor family
TRIF: TIR-domain-containing adaptor-inducing interferon-β
Tyk: Tyrosine kinase
WNV: West Nile virus
YFV: Yellow fever virus
